# A Short Review on Minimum Description Length: An Application to Dimension Reduction in PCA

**DOI:** 10.3390/e24020269

**Published:** 2022-02-13

**Authors:** Vittoria Bruni, Maria Lucia Cardinali, Domenico Vitulano

**Affiliations:** 1 Department of Basic and Applied Sciences for Engineering, Sapienza Rome University, Via Antonio Scarpa 16, 00161 Rome, Italy; vittoria.bruni@uniroma1.it (V.B.); marialucia.cardinali@uniroma1.it (M.L.C.); 2 Istituto per le Applicazioni del Calcolo, Via dei Taurini 19, 00185 Rome, Italy

**Keywords:** minimum description length, principal component analysis, dimension reduction, classification, features extraction

## Abstract

The minimun description length (MDL) is a powerful criterion for model selection that is gaining increasing interest from both theorists and practicioners. It allows for automatic selection of the best model for representing data without having a priori information about them. It simply uses both data and model complexity, selecting the model that provides the least coding length among a predefined set of models. In this paper, we briefly review the basic ideas underlying the MDL criterion and its applications in different fields, with particular reference to the dimension reduction problem. As an example, the role of MDL in the selection of the best principal components in the well known PCA is investigated.

## 1. Introduction

Dimensionality reduction plays a crucial role in the analysis of high-dimensional data. It consists of reducing the number or the dimension of features referred to in a given class of data without losing the capability of being distinctive for that class. It represents a critical issue in classification, as it has been widely proved that classifiers are not able to reach their goal whenever the number of features is too high (too much data) or too small [1,2]. The literature is rich in methods and approaches for reaching this goal; some well-known and popular examples are principal components analysis (PCA) [3], non-negative matrix factorization [4], isomaps [5], t-distributed stochastic neighbor embedding [6], uniform manifold approximation and projection for dimension reduction [7], autoencoders [8], multidimensional scaling (MDS) [9], and so on. Each one is based on one or more criteria to use for dimension reduction. For example, principal components analysis (PCA), which represents one of the most popular and commonly used methodologies, mainly consists of an orthogonal projection of the data onto a lower-dimensional linear space, where the variance of the data is preserved or maximized. As a matter of fact, the dimension reduction problem resembles the sparsity problem, as it requires condensing the peculiarity of the object of interest, which makes it distinguishable from others, into a very small number of features. This comparison/connection is as true as those features that are the coefficients of a given transform: linear or not linear, redundant or not redundant, defined by a single basis or a dictionary. However, the data compaction/compression task is a longstanding, still unsolved, and open problem that has been partially overcome by introducing a dictionary of bases. However, even in this case, it is necessary to define a fast and effective algorithm for the selection of the most significant elements of the dictionary.

The equivalence between dimension reduction, sparsity, and optimal coding tasks, especially in the blind context, is well summarized and conveyed by the minimum description length (MDL) principle, which allows the selection of a good model for approximating the data with the least complexity [10]. It is based on the concept that good compression means good approximation, which is in agreement with the Kolmogorov complexity.

The MDL principle was formulated about 30 years ago [10,11]. It is mainly based on information theory principles and has inherited several aspects from the Kolmogorov complexity [12]. It has been designed as a statistical inference method [13], where the rationale is that the observed data have to be compressed by the model. Several candidate models can be then compared on the basis of how much they can compress data—by retaining useful information while discarding noise. It turns out that the best model (i.e., the model along with its free parameters) will be the one that gives the shortest code for the data under examination [14,15]. It is worth outlining that MDL changes the perspective of model selection. In fact, it does not assume any ’true’ model for the specified data, as do classical probability and Bayesian models. It simply tries to do its best with the set of available candidate models. The basic principle founding MDL is, then, very simple: the simplest model that fits the data well is also the best one.

The simplest formal way to implement MDL is the crude MDL (or two-part code); it selects a model from a set of candidates by minimizing the total cost that is defined as the cost (expressed in terms of bits) required for coding the model plus the number of bits required for coding the data given the model. It is worth observing that the latter is strictly related to the ability of the model to represent the data, and it is often reached by costly models. Hence, the selection of the best model consists of a trade-off between the complexity of the model and good data representation/coding. Unfortunately, the practical construction of the MDL functional is not trivial, nor is its minimization. This is why the literature is rich in proposals that allow researchers to address one or more of these technical issues. In any case, despite the difficulty in designing effective and computable algorithms, many papers demonstrate, both theoretically and empirically, that the information-theoretic minimum message length principle has some advantages over the standard maximum likelihood estimate [16,17]. In addition, as proved in [18,19], a robust, monotonically convergent, and moderately short algorithm for the selection of the optimal two-part MDL code can be defined only by taking advantage of the concept of Kolmogorov complexity.

MDL was originally designed for model selection (see, for instance, [13,20,21,22,23]) and it has been successively applied in different contexts and for different tasks, such as, for example, picking and tuning the best parameters for a given model [14,15]. However, as mentioned at the beginning of this section, in this paper, we are mainly interested in focusing on the feature reduction problem and, specifically, on how MDL can help with ’automatically’ setting the number of components in PCA [24,25,26]—this represents one of the widely used tools for dimension reduction. To this end, the approximation of the normalised version of the MDL functional proposed in [27] has been studied and applied to some conventional data classification problems.

The remainder of the paper is organized as follows. The next section reviews the theoretical formulation of MDL principle. Section 3 presents a brief overview of some of the main uses of the MDL principle in the field of data processing, with a particular focus on principal component analysis. To show the advantages and potentialities of this framework, some numerical examples concerning MDL-PCA are presented in Section 4, with reference to data classification. Finally, Section 5 draws the conclusions.

## 2. A Short Review about MDL

The interest of the scientific community has been increasing in recent years, and different versions of MDL have been proposed. In the following, a short overview of the crude MDL (sometimes dubbed ’two-part’ code) will be given. However, the normalized maximum likelihood (NML) is actually the most-adopted version of MDL, as it provides an effective solution that is supported by an elegant formalism. Hence, a short review of NLM will be provided. The following explanation is not exhaustive at all. For further reading, an introductory and simple lecture on NML can be found in [13], while a technical summary can be found in [21,28,29]. Details concerning how NML can be used to select the optimal number of features in a classification problem will be given in the next section. Specifically, the approach in [27] will be presented and discussed in order to evaluate pros and cons of NML as a ’features reduction device’.

As already outlined, the simplest formal way to implement MDL is the crude MDL. Specifically, the simplest model that fits a given data sample x well is also the best one. It turns out that the best MDL model $\bar{M}$, from a set of candidates $M^{\left(1\right)},M^{\left(2\right)},\cdots$ is given by the following minimization:

(1) $$\bar{M}=\underset{M=M^{\left(1\right)},M^{\left(2\right)},\cdots }{\mathrm{argmin}}l\left(M\right)+l\left(x|M\right),$$

where l(M) is the cost (in terms of bits) for coding the model M, and l(x|M) is the cost required for coding the data x given the model. This minimization is not trivial, as it leads to the best trade-off between two competing requirements: the approximation performance of the model and its cost—measured in terms of bits. The result will then be a suitable balance between (model) complexity and (data through the model) representation.

### From Crude MDL to Refined MDL: NML

Crude MDL can be considered the first implementation of the Rissanen philosophy. However, its use is limited in different applications, as it usually needs a suitable weight λ for balancing the two cost terms that define the functional minimized in Equation (1). In fact, as emphasized in [21], ”it is more problematic to find a good code for hypotheses M and often ‘intuitively reasonable’ codes are used; however, it can happen that the description length l(M) of any fixed point hypothesis *M* can be very large under one code, but quite short under another”, making the procedure somewhat arbitrary. There are several approaches in the literature that use crude MDL in a empirical way by applying a corrective weight to one of the lengths in Equation (1), or by properly selecting the coding procedure [15,30,31]—even with optimal performance. A way of making MDL perform better while still being elegant consists of its refined version, namely, the normalized maximum likelihood (NML). In order to introduce this, some preliminary information theory concepts have to be recalled.

In information theory, it is well known that any message x, i.e., a sequence of *n* symbols $x_{1},x_{2},\cdots x_{n}$ belonging to a binary alphabet H={0,1}, can be encoded and compressed in a new message y with *m* symbols with m≤n — as matter of fact, there is no constraint on the alphabet: the only request is a finite cardinality, and the binary one allows us to denote messages’ lengths in terms of bits. In other words, message x can be compressed, giving the (possibly) shorter message y. More formally, we can say that the codelengths of x and y satisfy the following relation, l(x)≥l(y), where *l* is the codelength function. There are various ways to encode x. A property required for the codewords that have to encode x is that they belong to a prefix code: any codeword must not be a prefix of any other. This requirement is necessary to produce a uniquely decodable code that is also instantaneous: any codeword can be decoded once it has been received [12]. This class of (prefix) codes plays a fundamental role, as it regulates the foundations of information theory. On the one hand, there is a sort of equivalence between probability distributions and prefix codes. For any probability density function (pdf) *p*, there is a corresponding prefix code able to encode x via a code with length $l\left(x\right)=-\lceil \log_{2}p\left(x\right)\rceil$, where ⌈·⌉ denotes the first integer ≥·. On the other hand, Shannon’s source coding theorem states that this code, with its relative (ideal) length l(x)−logp(x), is optimal for x and then for *p*. To make the presentation more general, in this paper, log will be used by considering a generic basis. From an Information Theory point of view, $\log_{2}$ should be used in agreement with the only language any real device understands: the binary one.

Now, let us suppose that we have a family M of distributions f(·|θ) depending on one or more parameters θ. Let us suppose that we have to select among them the model that better fits x. Obviously, the best approximation, i.e., the best code for any data x, will be $f(\cdot |\hat{\theta })$, where $\hat{\theta }$ is the maximum likelihood estimate for the data x. The codelength of the optimal encoding for x, using any distribution of M, is called the *stochastic complexity* of x with respect to M. However, the optimal encoding of x, using $f(\cdot |\hat{\theta })$, cannot be used in practice, as its code cannot be specified before data observation. It turns out that an alternative strategy must be found. In particular, the idea is to determine a distribution, and then a code, that performs as well as the family of distributions belonging to M [20]. MDL suggests introducing the concept of universal distribution $p_{u}$, formally defined as:

$$-\log\left(p_{u}\right)\left(x\right)\leq -\log(f\left(x|{\hat{\theta }}_{x}\right)+C_{n}\left(M\right),$$

where $C_{n}\left(M\right)$ behaves as o(n), i.e., $lim_{n}\frac{C_{n}\left(M\right)}{n}=0$. The corresponding code will be denoted with universal code. $C_{n}\left(M\right)$ characterizes the universal distribution and represents the maximum difference between the two codes (the universal and the maximum likelihood ones). It is worth observing that more than one universal distribution may exist, and each one is characterized by its own $C_{n}\left(M\right)$; however, apart from a constant, they are almost equivalent and good to approximate x.

As already outlined, NML does not assume a ’true’ distribution. It simply requires a distribution that is able to fit the data, separating useful information (to preserve) from noise (to discard) [32]. Keeping in mind that, theoretically, the best the model family can do on a dataset x is $-\log f(x|{\hat{\theta }}_{x})$, but this is useless from an information theory point of view because it requires prior knowledge of the data x; the universal distribution $p_{u}\left(x\right)$ allows us to write the additional cost required to encode it, as it follows:

$$-\log p_{u}\left(x\right)+\log f\left(x|{\hat{\theta }}_{x}\right).$$

This quantity is called ’regret’ of p(x) with respect to M with the data x. It expresses the fact that the universal distribution $p_{u}\left(x\right)$ works well, but not ’perfectly’, as the original (hypothetical) distribution that originates data x does not exist. Now, the natural question is: which is the worst case scenario?

It can be simply written as a regret, and then:

$$R(p_{u}\left||M\right)=\max_{q}E_{q}\left[\log\frac{f(x|{\hat{\theta }}_{x})}{p_{u}\left(x\right)}\right]\;\forall q.$$

Such a regret should hold for any distribution *q*, while $E_{q}$ is the relative expectation. The expression above recalls the Kullback–Leibler divergence [12] that was originally designed for measuring ’the extrabits’ required when a message is encoded through a ’wrong’ distribution. Hence, the problem can be seen as a minimax one, and it can be formalized as follows:

$$f^{NML}=\arg_{p}\min_{p}\max_{q}E_{q}\left[\log\frac{f(x|{\hat{\theta }}_{x})}{p(x)}\right].$$

It is worth providing a few additional details about the equation above.

*q* stands for any probability distribution that guarantees that $E_{q}\left[\log\frac{q(x)}{f(x|{\hat{\theta }}_{x})}\right]$ is finite. $f^{NML}$ is then the NML solution, and it is defined as [20,33]:

(2) $$f^{NML}=\frac{f(y|{\hat{\theta }}_{x})}{\int f(y|{\hat{\theta }}_{y})\;dy}.$$

It is straightforward to see that ${\hat{\theta }}_{y}$ is the ML for the data x. Moreover, the denominator integrates over the ML of all possible datasets in a specified context (the one that originated x). The corresponding codelength $-\log(f^{NML}\left(x\right))$ is the stochastic complexity of x with respect to M, that is,

$$sc\left(x\right)=-\log f\left(x|{\hat{\theta }}_{x}\right)+\log\int f\left(y|{\hat{\theta }}_{y}\right)dy.$$

It is worth outlining that it is not required for $p_{u}$ and *q* to belong to the model *M* as well as $f^{NML}$—an interesting example is in [13], where the selected distribution does not behave as the one that originated the data.

The stochastic complexity is composed of two terms: the first one quantifies how much the model *M* approximates the data x, while the second one is a measure of the complexity of *M*. The latter is interesting, as it describes ’how many data’ can be well fitted by the model *M*: as many data can be fit by *M* as the model *M* is complex [34].

Finally, an equivalent definition of complexity is provided by the minimum of the worst-case expected regret—details can be found in [33]. Additional aspects concerning MDL, such as asymptotic approximations to NML, and its relation to Bayesian statistics and MDL predictive inference, are out of the scope of this contribution—a deeper but simpler reading concerning these topics can be found in [13].

## 3. MDL Applications: A Review

Despite the difficulty of its practical application and implementation, MDL has been widely used in different fields by introducing different kinds of approximation, technical tricks, bounds, and so on, for practically using and adapting it to each context. Rissanen himself accurately studied the problem of MDL-based denoising and clustering. In the first case, noise is considered the incompressible part of the data [35]; as a result, MDL can provide the best threshold value whenever denoising is performed in the wavelet domain. In the second one [36], optimal clustering provides the best compression, i.e., the lowest coding cost for each cluster. In this section, we briefly describe some examples of the variety of applications and uses of MDL by grouping them with respect to the main purpose of the specific application they refer to.

Most of papers concerning MDL mainly use it according to its general and original meaning, i.e., the compression and learning model. In this context, it is worth mentioning some recent studies which provide new practical MDL-based ways to compute tight compression bounds in deep-learning models. In particular, in [37], it has been observed that prequential coding yields much better codelengths than variational inference, correlating better with the test set performance — we remind that in the prequential coding, a model with default values is used to encode the first few data; then, the model is trained on these few encoded data; the partially trained model is used to encode the next data; then, the model is retrained on all data encoded so far, and so on. On the contrary, in [38], an MDL-based strategy is used for determining a parameter-free stopping criterion for semi-supervised learning in time series classification, while in [39], the problem of model change tracking and detection has been addressed and studied in both data-compression and hypothesis-testing scenarios. In the first case, an upper bound for the minimax regret for model changes has been found; in the second one, error probabilities for the MDL change test have been derived, and they rely on the information-theoretic complexity, i.e., the complexity of the model class or the model itself and the α-divergence. In a more recent paper [40], the same author introduced the descriptive dimension that characterizes the performance of the MDL-based learning and change detection. In the context of machine learning, MDL has been used for preventing overfitting [41], especially in the case of little available training data. In particular, it has been used for ensuring that there is less information in the weights than in the output vectors of the training cases; to this aim, the model cost is the number of bits it takes to describe the weights, and the cost of the data given the model is the number of bits it takes to describe the discrepancy between the correct output and the output of the neural network on each training case. Very recently, in [42], the neural network training process has been seen as a model selection problem, and the model complexity of its layers has been computed as the optimal universal code length by means of a normalized maximum likelihood formulation. This kind of approach offers a new tool for analyzing and understanding neural networks while speeding up the training phase and increasing the sensitivity to imbalanced data. More generally, model selection theory allows for an information-theoretic analysis of deep neural networks through the information bottleneck principle [43,44].

With regard to fitting/regression, in [45], MDL is used to successfully reduce the number of false positives in best-fitting-based gene regulatory networks that govern specific cellular behavior and processes. In particular, it has been proved that MDL-based filtering strategies can be computationally less burdensome than using the MDL algorithm alone; in fact, the computation of data-coding length is more complex than calculating the error estimate of the best-fit algorithm, and the computational complexity increases dramatically as the sample size increases. In the same application context, MDL is used for finding the optimal threshold that defines the regulatory relationships between genes [46]. In a different context, and using a different strategy, MDL is used for determining the number of modes in non stationary and highly oscillating signals [31], while in [47], MDL allows for unsupervised spectral unmixing of spectrally interfering gas components of unknown nature and number.

A pioneering paper concerning MDL-based clustering is [48], where a simple MDL cost functional is used to search the tree for a level of clustering with a minimum description length. In [36], the MDL principle is used for data clustering based on the assumption that a good clustering is such that it allows efficient compression when the data are encoded together with the cluster labels. It is worth stressing that, based on the observation that an efficient compression is possible only by discovering the underlying regularities that are common to all the members of a group, this approach also implicitly defines a similarity metric between the data items. Formally, the global code length criterion to be optimized is defined by using the intuitively appealing universal normalized maximum likelihood code, which has been shown to produce optimal compression rates in an explicitly defined manner—the local independence of the model has to be assumed to get a computable algorithm. Ref. [49] presents a study concerning the use of MDL, specifically, the normalized maximum likelihood (NML) version, in the dynamic model selection. The aim is to track changes of clustering structures so that the sum of the data’s code-length and clustering changes’ code-length is minimized. The study is restricted to the Gaussian mixture model for representing the data, and it has been shown that the proposed method is able to detect cluster changes significantly more accurately than the Akaike information criterion (AIC)-based methods [50] and Bayesian information criterion (BIC) [51]-based methods—an application to market analysis is proposed. In [52], MDL is used for IoT applications. Specifically, a hierarchical clustering is applied for grouping datasets received from sensor nodes: if any pairs of received datasets can be compressed by the MDL principle, they are combined into one cluster.

MDL based strategies are successfully applied for solving the dimension/features reduction problem. Among them, it is worth mentioning the one recently presented in [27], where MDL has been used for the selection of the number of components for the PCA method. Since it is not trivial to practically define MDL, a linear regression model has been used as the bound for its normalized version. In the same context, MDL-based matrix factorization has been proposed in [53], where the objective function is designed through an MDL-based formulation to guide the formation of the matrices defining the model, allowing an automatic and natural trade-off between accuracy and model complexity. In [54], the problem of finding the appropriate feature functions and number of moments is formulated as a model selection problem. MDL is then used for solving it, and it has also been shown that it generalizes the minimax entropy principle. The method has been successfully applied to the gene selection problem to decide on the quantization level and number of moments for each gene; however, the extension to problems involving larger datasets requires more efficient approximations to calculate the complexity.

As further examples, MDL can also be properly used for selecting: (*i)* the least number of image points from which image quality is assessed, in agreement with the human visual system information coding approach, as in [30]; (*ii*) features, which are selected adaptively during online learning, based on their usefulness for improving the objective function, as in [55]; (*iii*) points on shapes defined as curves to allow for shape recognition, as in [56]; (*iv*) a characteristic subset of patterns on labeled graphs with complex shapes and that are representative of the data, as in [57]. Interesting MDL applications are also the ones that directly work in the wavelet domain and that further take adavantage of the data decorrelation and compaction properties of the transform. For example, in [58], the MDL principle is used for preventing over- or underfitting problems in detrending near-infrared spectroscopy (NIRS) data for neuroimaging applications; in [59], the same principle is used for wavelet-based compression and, in particular, for the selection of the best wavelet and threshold, while in [60], the soft-thresholding-based denoising problem is considered. Finally, in [61], the noisy and original data are properly separated by determining their histogram and retaining the coefficients belonging to specific bins—the optimal set of bins is found by minimizing the sum of the two code lengths for the denoised signal and the noise.

Finally, with regard to the computational cost required by the implementation of an MDL-based method, several efforts have been made in the literature. As a representative example, we mention the method presented in [62], where a computationally feasible algorithm for computing the NML (normalized maximum likelihood) criterion for tree-structured Bayesian networks has been proposed. In particular, the exponential time, required for building Bayesian trees and forests, has been reduced to a polynomial law—in this way, the advantages offered by the information-theoretic normalized maximum likelihood (NML) criterion in Bayesian network structure learning are preserved and easily exploited.

### NML for Dimension Reduction in PCA

As mentioned in the Introduction, in this paper, we focus on MDL-based feature reduction and, in particular, on the ’automatic’ selection of the number of components in PCA (principal component analysis). The standard measure of quality of a given principal component is the proportion of total variance that it accounts for. As a result, very often, the desidered percentage is fixed and the number of components is derived. However, the number of components often depends on the specific task, and setting the optimal percentage of variance to retain is sometimes user-dependent. However, as the problem is crucial, different methods and criteria have been proposed in the literature. A possible classification of those methods refers to the methodological approach [63,64], i.e.:

- ad-hoc rules, as, for example, the Cattel’s scree test [65] and the indicator function [66];
- statistical tests, such as Bartlett’s test [67] and the Malinowski’s F-test [68];
- computational criteria, such as cross-validation (CV) [69], bootstrapping and permutation, such as Horn’s parallel analysis [70], and SVD-based methods [71].

However, it has been shown that each selection method performs differently in real cases, depending on the task. In addition, most of them require a certain computational burden—see [69] for a complete review.

An interesting approach that combines NML and PCA is contained in [27], where an elegant formulation for solving this problem is proposed.

Let us suppose that X is an n×m matrix, containing the data or the corresponding features. The PCA of X consists of the following minimization:

(3) $$\arg_{W,Z:rank(W)=rank(Z)}\min||X-WZ^{T}{\left|\right|}_{F}^{2},$$

where W and Z are two matrices whose sizes, respectively, are n×k and k×m, and whose rank is equal to *k*, while $\left|\right|\cdot {\left|\right|}_{F}$ denotes the Frobenius norm [72]. The following theorem [25,73] holds that:

**Theorem** **1** (Eckart–Young–Mirsk). *Let $\;X=U\Lambda V^{T}$ be the SVD (singular value decomposition) of X, with $\Lambda =diag(\lambda_{1},\cdots ,\lambda_{m})$, while U and V are unitary. Let $U_{k}$ and $V_{k}$ be the ’reduced versions’ of U and V, i.e., containing their first k columns, then:*

(4) $$||X-{\mathrm{WZ}}^{T}{\left|\right|}_{F}^{2}\geq ||X-U_{k}diag\left(\lambda_{1},\cdots ,\lambda_{k}\right)V_{k}{\left|\right|}_{F}^{2}=\sum_{i=k+1}^{m}\lambda_{i}^{2},$$

*with $W=U_{k}diag\left(\lambda_{1},\cdots ,\lambda_{k}\right)$; $Z=V_{k}$.*

This theorem shows that any ’selection/reduction’ component leads to a loss of information, and it also quantifies this loss. It turns out that, in principle, Equation (4) could be combined with the NML solution in Equation (2) in order to get the formal stochastic complexity of the PCA-based reduction of X to *k* components. Unfortunately, the evaluation of the denominator in Equation (2) is not trivial, as it depends on the eigenvalues of arbitrary matrices. The approach presented in [27] suggests a way to address this issue by adopting the NML of linear regression that takes advantage of quantized versions of the unitary matrices $V_{k}$. The main trick of this approach consists in considering the generative form of PCA, i.e.,

$$X=W_{k}V_{k}^{T}+\eta ,$$

where $\eta ∼N(0,\tau I_{k})$ is the error that is supposed to be normally distributed, and by considering a perturbation of the matrix $V_{k}$ as follows:

$$V_{k}^{\epsilon }=V_{k}+\epsilon E_{k},$$

where ϵ is the quantization bin size for the values of the unitary matrix $V_{k}$ (whose elements belong to the range (−1,1)), with $\epsilon \leq \frac{1}{m}$ and $|E_{k}|\leq \frac{1}{2}$, and by writing the corresponding NML—see [27] for the technical details. It turns out that the problem resembles the linear regression one, where the elements of the unitary matrix V are suitably quantized using the quantization parameter ϵ. This way leads to the following result, in agreement with [35]:

**Theorem** **2.** *Let sc(X;k) be the stochastic complexity of the PCA-based reduction of X to k components, then:*

(5) $$\begin{array}{cc} sc(X;k)≃ & \left(nm-kn\right)\log\left(\sum_{i=k+1}^{m}\lambda_{i}^{2}\right)+nk\log(||X^{T}{X||}_{F}^{2})+\\ & +\left(mn-kn-1\right)\log\left(\frac{mn}{mn-kn}\right)-\left(nk+1\right)\log\left(nk\right)+\Delta s, \end{array}$$

*with 0≤Δs≤mklog(2/(mϵ)), n×m as the dimension of X, and ϵ as the quantization bin, such that $\epsilon <\frac{1}{m}$.*

It is worth observing that the first term in the second member of Equation (5) represents the code length of the part of the data that adds no further information about the optimal model, i.e., the information that can be neglected; the remaining terms define the length of a code from which the optimal model, which is defined by the ML parameters and that belongs to the subclass of quantized loading matrices of rank *k*, can be decoded. As a result, the optimal number of principal components is the value of *k* that minimizes the second member of Equation (5), and the latter only depends on quantities that are known or that can be computed directly from the data.

## 4. Experimental Results

To better evaluate pros and cons of the theoretical results presented in the previous section, three numerical experiments are presented, referring to two very different datasets. The first dataset is the hyperspectral image Indian Pines [74], captured through the AVIRIS sensor at the Indian test site of North-Western Indiana; each spectrum contains the spectral information of 220 bands in the 0.4–2.5 μm wavelength region, and it is classified in one of the 16 (+ background) identified classes (such as farmland, forest, highway, housing); each image is composed of 145×145 pixels. In particular, the corrected Indian Pines dataset has been downloaded, in which the number of spectral bands is reduced to 200 by removing the ones covering the region of water absorption. The second dataset consists in 162 ECG recordings from the PhysioNet database [75] obtained from three groups of people with cardiac arrhythmia (96 records), congestive heart failure (30 records), and normal sinus rhythms (36 records). For comparative studies, two standard methods for the selection of the number of components to be retained have been considered. The first one refers to the percentage of variance that the components are required to retain; in particular, 90%, 95%, and 99% of the variance of the original data have been considered; the second one refers to the Bartlett test [67]. All tests have been performed implementing a Matlab code (release 2021) on a Intel(R) Core(TM) i7-1065G7 CPU 1.30GHz-1.50 GHz workstation with RAM equal to 16GB.

For the sake of clarity, we split the formula of sc(X;k) in Equation (5) into the following terms:

1. $a=\left(nm-kn\right)\log\left(\sum_{i=k+1}^{min(m,n)}\left[\lambda_{i}^{2}\right]\right)$;
2. $b=nk\log\left(\left|\right|X^{T}X{\left|\right|}_{F}^{2}\right)$;
3. $c=\left(mn-kn-1\right)\log\left(\frac{mn}{mn-kn}\right)$;
4. d=−(nk+1)ln(nk);
5. $e=mk\log\left(\frac{2}{m\epsilon }\right)$, i.e., the upper bound of Δs.

The value of the quantization parameter ϵ has been selected using the theoretical results concerning high resolution quantizers [76]. In this context, the distortion is minimized with a uniform scalar quantization, which means that the distortion has to be significantly less than the variance of the signal to quantize [77]. That is why ϵ has been selected two or three orders of magnitude less than the variance of the matrix V of the SVD decomposition of original data matrix X.

**Figure 1:**
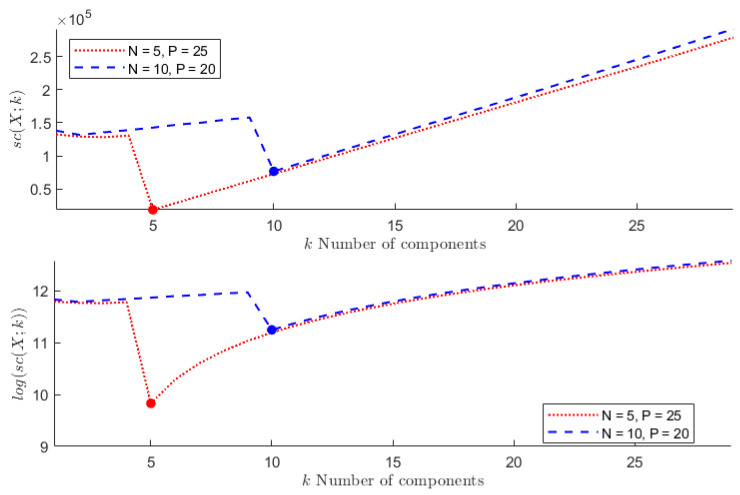
TEST 1. (**Top**) Red dotted line: sc(X;k) versus the number of components *k* for N=5, P=25; the minimum is correctly attained at k=5. Blue dashed line: sc(X;k) versus the number of components *k* for N=10, P=20; the minimum is correctly attained at k=10. (**Bottom**) The same plot where log(sc(X;k)) has been considered to improve its readability in correspondence to the minimum value.

**Figure 2:**
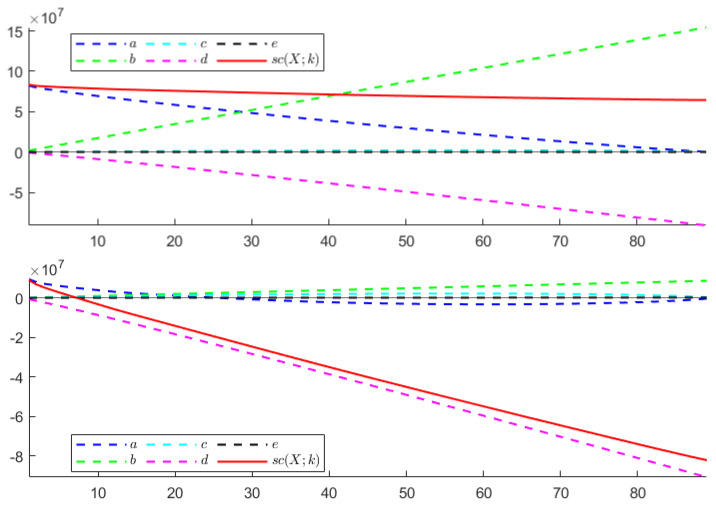
TEST 2. Plot of sc(X;k) and its components versus the number of components *k*. (**Top**): non-normalized data. (**Bottom**): normalized data.

**Figure 3:**
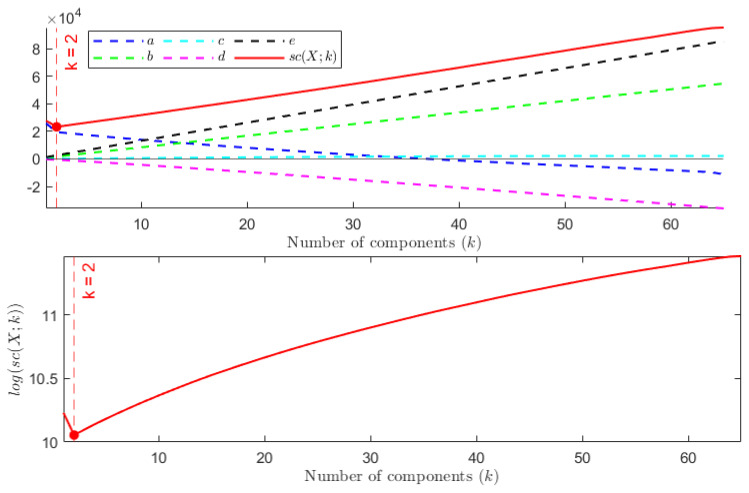
TEST 2. (**Top**) Plot of sc(X;k) and its components versus the number of components *k*. Signals have been uniformly sampled so that the dimension of X is n×m=66×90. (**Bottom**) Plot of log(sc(X;k)).

**Figure 4:**
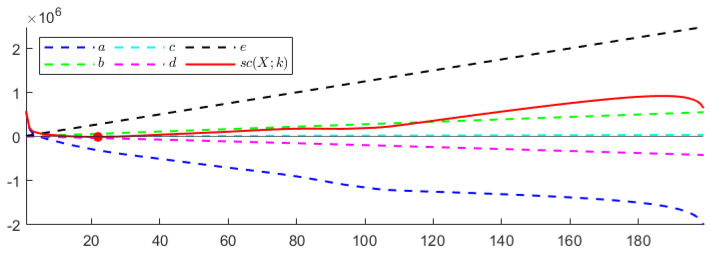
TEST 3. Plot of sc(X;k) and its components versus the number of components *k*; the minimum is attained at k=22.

**Figure 5:**
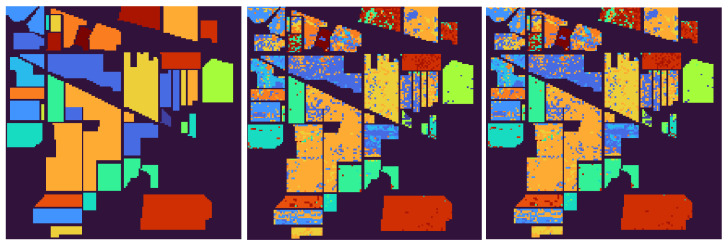
TEST 3. (**Left**) Ground-truth Indian Pines image; (**Middle**) classification image using the best result of PCA-SVM in [78]; (**Right**) classification image using the PCA-SVM method and the number of components estimated using the stochastic complexity, as in Equation (5).

**TEST** **1** The first test is carried out on the hyperspectral dataset and follows the numerical experiments presented in [27]. A set composed of *N* signals randomly picked from *N* different classes (N≤16) plus *P* random linear combinations of them corrupted by Gaussian noise has been considered—the weights of the linear combination are extracted from a normal distribution of non-negative values with variance $\sigma^{2}=1$, while the Gaussian noise is zero mean, with standard deviation equal to σ=0.001. The goal is to find the number of the original signals *N*.
  Each column of the resulting matrix X is a signal so that the dimension of X is n×m, with n=200 being the number of spectral bands and m=(N+P) being the total number of signals. In agreement with [27], the following two configurations have been considered: *(i)* N=5 and P=25, *(ii)* N=10 and P=20. In both cases, the number of independent components *N* is correctly identified. Figure 1 depicts the behaviour of sc(X;k) with respect to *k*. As can be observed, the estimated stochastic complexity clearly presents a minimum in correspondence to k=N. It is worth noting that the local relative minimum shown by the two curves is caused by the term *a*, which depends on the singular values. The quantization step ϵ has been set equal to $10^{-8}$. However, it is worth noting that, in this case, the choice of the quantization parameter is not crucial, since the contribution of the term *e* to the general trend of sc(X;k) is negligible when compared with the contribution of the term *b*. The computing time required for performing the test has been less than 0.066 s.
**TEST** **2** The second test refers to ECG data. Here, the same number of signals is randomly selected from the three classes, and the aim is to identify the number of classes.
  It is worth observing that, in this case, the dimension of the data matrix X is such that m≤90, while n=65,536. As a consequence of this imbalance, the combined effect of the terms *a* and *d* for not-normalized data, and of the term *d* in the case of data normalized w.r.t the (euclidean) norm of the signal with a maximum norm, leads to a trivial absolute minimum corresponding to k=m, independently of the choice of the quantization step ϵ—resulting in a not-consistent estimation of the cost of the model. This results in the conclusion that the formula in Equation (5) generally fails in any case for which the length of the signals *n* far outweighs their number *m*. In Figure 2, the shape of sc(X,k) is depicted for both not-normalized and normalized data, $\epsilon =10^{-8}$, and m=90 (30 recordings from each class). Similar plots are obtained for m=60 and m=30. The computing time required for performing the test has been less than 0.135 s. More consistent results are obtained by sampling the analyzed signals; however, sampling may cause the loss of some distinctive features for the signal belonging to the different classes, resulting in the estimation of a smaller number of independent classes, as is shown in Figure 3. In this case, a NML depending on both the number of components and the sampling step would be preferable.
**TEST** **3** The third test aims at using the proposed NML-based feature reduction method in a more interesting (real) case concerning hyperspectral image classification.
  For classification purposes, the conventional approach consists of first reducing the dimensionality of the data by applying PCA, and then feeding the transformed data to an SVM (support vector machine), which classifies them. It is straightforward that the selection of the right number of new components is a core problem, and often, several trials are needed to find the best classification score, resulting in a time-consuming and computationally expensive process.
  Our intent is to determine whether minimizing sc(X;k) allows us to simplify the process, i.e., if it could be a good choice to simply select the first $\hat{k}$ components, where $\hat{k}$ minimizes sc(X;k). For the numerical experiment, the procedure adopted in [78] is taken as a reference, and the results concerning the Indian Pines dataset are compared with the ones presented there. Accordingly, the training set for the SVM is composed of 10% of the samples in each class, randomly selected and normalized; these samples are the columns of the matrix X that is analyzed. As depicted in Figure 4, the value $\hat{k}$ which minimizes sc(X;k) is 22, which is consistent with the best classification result for PCA+SVM obtained in [78], as shown in Figure 5.
  In this case, the ϵ-dependent term *e* plays a key role in determining the trend of sc(X;k) for two reasons: first, the arguments of the logarithms in the terms *b* and *e* have the same magnitude; second, the dimensions *n* and *m* of the matrix X are such that n<<m, so that the term *e* overwhelms the term *b* as *k* grows. It turns out that, in this case, the selection of the quantization step ϵ is crucial. As in the first test, the presented results refer to $\epsilon =10^{-8}$ and the required computing time has been about 1.10 s.

To conclude this section, Table 1 contains the number of components selected using standard criteria for the selection of components in PCA, i.e., the percentage of the total variance and Bartlett’s test. The table refers to the three tests described above. As it can be observed, the MDL criterion is able to select the number of components closer or equal to the expected one in almost all tests, showing some robustness to the task. This is due to the fact that the MDL criterion tends to maximize the accuracy with the least cost. This confirms the potential of the MDL criterion in feature reduction procedures and offers a new and different approach to the solution of the selection of the best principal components’ number.

## 5. Conclusions

This short review has shown some of the main features of MDL by referring to a few specific applications. MDL is appealing in data approximation-based problems, as it simply uses available data and models to make the best choice. In fact, the rationale of discarding the hypothesis that a ’true’ distribution produced the current data is a conceptual step forward in data analysis. In addition, apart from the model selection problem, MDL has shown, in its different declinations, to be an effective tool for many other applications. The selection of the suitable number of features to adopt in the classification process is only the latest of the several applications where it plays a fundamental role. In addition, this specific use opens new possible ways in machine/deep learning that implicitly or explicitly depend on both the type and the number of features. On the other hand, as the presented simulations have shown, MDL often suffers from an explicit or implicit dependence on one or more parameters that have to be set. Usually, this is not a critical step, as setting them often is easier than competing approaches. However, this is one of the main points to be investigated in the future research.

## Figures and Tables

**Table 1:** Number of principal components selected for the three tests by using different criteria: percentage of variance to be retained (90%,95%,99%), Bartlett’s test with significance level α equal to 0.05 and 0.01, and the MDL criterion. The last column contains the expected number.

Test	90%	95%	99%	Bartlett’s Test (α=0.05)	Bartlett’s Test (α=0.01)	MDL	True Value
Test 1	1	1	1	30	30	5	5
Test 2	37	52	75	90	90	90	3
Test 2	2	6	24	63	63	2	3
(decimated data)							
Test 3	2	6	27	161	159	22	22

## Data Availability

Not applicable.
